# Respondent-driven sampling among gay and bisexual men: experiences from a New Zealand pilot study

**DOI:** 10.1186/s13104-015-1449-5

**Published:** 2015-10-09

**Authors:** Adrian H. Ludlam, Peter J. W. Saxton, Nigel P. Dickson, Jeffery Adams

**Affiliations:** Gay Men’s Sexual Health Research Group, Department of Social and Community Health, Faculty of Medical and Health Sciences, University of Auckland, Auckland, New Zealand; AIDS Epidemiology Group, Department of Preventive and Social Medicine, University of Otago, Dunedin, New Zealand; SHORE and Whariki Research Centre, College of Health, Massey University, Auckland, New Zealand

**Keywords:** Respondent-driven sampling, Gay and bisexual men, Sexually transmitted infections, HIV, Probability sample, Pilot study, Feasibility study, Formative assessment, New Zealand

## Abstract

**Background:**

Respondent-driven sampling (RDS) is a method of approximating random sampling of populations that are difficult to locate and engage in research such as gay, bisexual and other men who have sex with men (GBM). However, its effectiveness among established urban gay communities in high-income countries is largely unexplored outside North America. We conducted a pilot study of RDS among urban GBM in Auckland, New Zealand to assess its local applicability for sexual health research.

**Findings:**

Pre-fieldwork formative assessment explored RDS suitability among local GBM. Highly-networked initial participants (“seeds”) and subsequent participants completed a questionnaire, took a rectal swab for chlamydia and gonorrhoea testing, and were asked to recruit up to three eligible peers over the subsequent 2 weeks using study coupons. Compensation was given for participating and for each peer enrolled. Feedback on the pilot was obtained through questionnaire items, participant follow-up, and a focus group. Nine seeds commenced recruitment, directly enrolling 10 participants (Wave One), who in turn enrolled a further three (Wave Two). Two of the 22 participants (9 %) had undiagnosed rectal chlamydia. The coupon redemption rate (23 %) was lower than the expected rate (33 %) for this population. Participants were motivated by altruism above financial incentives; however, time, transport and reluctance recruiting peers were perceived as barriers to enrolment.

**Discussion:**

Slow recruitment in our pilot study suggests that RDS might not be an effective or efficient method of sampling gay men in all high-income urban settings. However those who participated in the pilot were willing to provide anal swabs and information on their sexual behaviour, and also on the size of their GBM social network which is necessary to weight data in RDS. Refinements and adaptations such as reducing the transaction costs of taking part (e.g. offering online participation) could improve responses but these have their own drawbacks (higher set-up costs, difficulty collecting biological specimens).

## Background

Respondent-driven sampling (RDS) is a method that is purported to approximate a random sample in populations that are difficult to locate and engage in research. It is a form of chain-referral sampling in which participants are also each asked to recruit a limited number of eligible peers. Importantly, RDS methods provide a way of overcoming biases inherent in chain-referral sampling, and therefore of approximating probability data, so long as recruitment protocols are followed, and participants’ data account for homophily and are inversely weighted based on peer network size [1, 2]. Because participation in RDS studies typically requires presentation to a physical study location, it also enables both biological specimens and behavioural data to be collected. Thus RDS has high potential for investigating HIV and sexually transmitted infection (STI) prevalence among groups such as gay, bisexual and other men who have sex with men (GBM) who are disproportionately affected.

In spite of the strong theory on which RDS is based, there is limited evidence that it has actually been a valuable method to research GBM across a range of settings. Anecdotally several attempts have been unsuccessful or have violated key assumptions [3]. Unusually, few RDS studies have been reported in high-income countries with established GBM communities in Western Europe or Australasia [4, 5], with the majority of successful studies undertaken in lower/middle income countries [6] or in North America [7–10]. This is despite the potential of RDS to provide superior estimates than the more commonly used gay community convenience sampling. Furthermore, Western European and Australasian countries generally have tolerant socio-legal environments towards homosexuality which should make RDS fieldwork comparatively simpler. Research examining the viability of RDS in these settings is needed.

New Zealand has an existing programme of non-random, purposive, community-based and web-based HIV behavioural surveillance among GBM [11], which has been used to collect oral fluid specimens [12]. However, collection of more comprehensive and invasive biological specimens for estimating STI prevalence among the GBM population, such as rectal specimens, is less feasible using these programmes. A quasi-probability sample derived through RDS, in which participants were willing to attend a centre and provide such specimens, would therefore be valuable.

The aim of this study was to conduct a pilot of RDS among urban GBM in Auckland, New Zealand, to assess whether this would be a suitable method of studying the prevalence of a range of STIs in this population.

## Methods

### Formative assessment

The pilot study was designed by adapting the methods of Johnston [13]. It was conducted in Auckland, a sprawling multicultural city of 1.4 million people. Auckland has the largest GBM population in New Zealand that is geographically clustered in the inner city [14]. Public and civic celebration of gay, bisexual, lesbian and transgender communities is common. A 2011 convenience-based study of GBM estimated HIV prevalence to be 6.5 % which was greater than among GBM living elsewhere in New Zealand [12].

Formative assessment with the community-based New Zealand AIDS Foundation (NZAF) sought feedback on the proposed method. NZAF also provided characteristics of social and sexual networks of GBM, and identified highly-networked initial participants (“seeds”). This consultation suggested that much of the GBM population in Auckland was networked through partially distinct “sub-tribes”, linked by overlapping members. It was agreed this would make an ideal environment for the use of RDS as almost all GBM would have a non-zero probability of being recruited, despite the possibility of sampling bottle-necks. The NZAF Burnett Centre, a community-based HIV-testing facility, was utilised for the study due to its central location, facilities to perform the study procedures, and public transport access.

### Seeds

Nine GBM from a range of ethnic groups (3 Maori, 2 Pacific, 2 Asian and 2 New Zealand European) with at least one in each group aged under and over 30 were selected as seeds to initiate recruitment. All seeds attended a motivational presentation on how RDS might improve their communities’ health if it proved to be a successful way of engaging GBM in research.

### Recruitment protocols

Seeds were asked to recruit eligible peers over a two-week period (Wave One), and those who they recruited asked to do the same over the following 2 weeks (Wave Two). Wave Two participants were not asked to recruit further peers in this pilot, but would be in a full study. Eligibility criteria were being male, aged 18 years or over, resident in Auckland, and having had sexual contact with another man in the past 5 years. No appointment was necessary to attend the study site. Study hours were Wednesday to Friday 11 am to 7 pm and Saturday 9 am to 5 pm. A part-time research assistant was employed to administer the study procedures on-site. Participants were required to provide a mobile phone number (which was destroyed at the conclusion of the pilot) for follow-up and to receive results if they tested positive.

Participants completed the study procedures on-site and were then provided with coupons—which had to be exchanged physically—to give to up to three eligible GBM they knew and who knew them. Coupons gave the study location, opening hours, contact number and brief information about the study. Movie vouchers, to the value of NZD 30 (approximately EUR 18), were provided on participation, with another for each peer successfully recruited (i.e. a maximum value of NZD 120/EUR 72). Alternatively, participants could ask that the equivalent be donated to a GBM-aligned charity. Each peer recruited by a participant and subsequently enrolled into the study was able to enjoy the same opportunity for earning vouchers for their own and others’ participation, and so on up to Wave Two. Therefore as with all RDS studies there was a dual incentive for participation: individual reward but also altruism towards peers or peer-organisations.

### Questionnaire

Questionnaires were self-completed on-site and deposited into a secure box. Questions included socio-demographics characteristics, sexual behaviour in the previous 6 months and the number of GBM they knew in Auckland, and their experiences of the study process.

### Specimen collection

We sought rectal specimens since GBM are at heightened risk of rectal STIs that are frequently asymptomatic. Rectal specimens are also difficult to collect in traditional convenience-based research settings such as at community events. Participants were asked to self-administer a rectal swab on-site using a Roche Corbas swab kit. Specimens were anonymously linked to participants by the respondent ID and sent to a laboratory for nucleic acid amplification testing (NAAT) for *Chlamydia trachomatis* and *Neisseria gonorrhoea*. Positive results were communicated to participants by the research assistant.

### Evaluation

Three ways were used to solicit feedback about the pilot study. Firstly, the questionnaire asked participants how easy it was to get to the study location, their chosen mode of transport, preferred access hours, motivation for taking part, the compensation amount, and whether they would be prepared to take part if participation wasn’t anonymous. Secondly, on redemption of coupons, both the seeds and Wave One participants were asked about people who declined to participate and their perceived reasons for doing so. Thirdly, a post-study focus group was conducted by an independent researcher with some seeds and Wave One participants. We selected a range of participants for this including those who had successfully recruited peers and those who had not. The focus group explored their understanding of the study process, compensation, site access, and barriers to participation. We also calculated the coupon redemption rate (number of coupons redeemed/number of coupons allocated to participants), with a rate of 33 % considered satisfactory for this population [13].

Ethics approval was received from the University of Otago Human Ethics Committee (Health)—#H13/010.

## Results

**Fig. 1:**
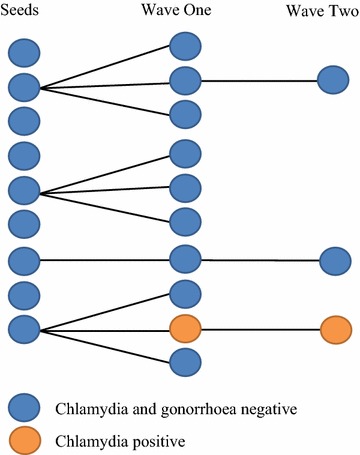
Diagram of RDS pilot study recruitment and results of rectal STI screening

Overall 22 participants were recruited into the pilot over 4 weeks (Fig. 1). The nine original seeds enrolled 10 participants; three recruiting the maximum of three peers within the 2-week limit, one recruited one and five none. These 10 in turn enrolled a further three; one each by three. This gave a coupon redemption rate of 23 % (13/57 coupons issued to participants to disburse to their eligible peers). Wave One and Two participants most commonly described their relationship to their recruiter as a “friend” (n = 12), than “previous sexual partner” (n = 3) or “current sexual partner” (n = 2) (multiple responses allowed). Two respondents (9 %) were diagnosed with rectal chlamydia, one having recruited the other and naming that person as a current sexual partner.

All respondents answered the peer network question (“about how many gay or bisexual men do you know in Auckland?”) with a mean of 172 and range of 10–2150.

Nineteen participants (86 %) reported little difficulty accessing the study site (Table 1). A third (32 %) preferred visiting the study site in the weekday evenings, and half (50 %) on a Saturday. The majority (86 %) said the most important motivator was “helping out with research for my community”, and most (82 %) that the compensation value was “about right”. Two-thirds (68 %) would agree to provide their names if this allowed for more comprehensive STI testing.

**Table 1:** Participant responses to RDS pilot study evaluation questionnaire

Questionnaire items relating to study evaluation	n (%)
How easy was it for you to get here today?	
Easy	14 (64)
OK	5 (23)
Not easy	2 (9)
Difficult	1 (4)
When would be the most convenient time to have come here?^a^	
Anytime	4 (19)
Weekday afternoons	1 (4)
Weekdays after 5 pm	7 (32)
Saturday	11 (50)
What is the most important reason for you taking part in this study?	
The payment, for me	3 (14)
The payment, for charity	0 (0)
Helping with research in my community	19 (86)
The anal swab	0 (0)
Thinking about the payment for participating…	
It should be more	1 (4)
It’s about right	18 (82)
It could be lower	3 (14)
Would you be prepared to take part in a study like this if we recorded your name, if it meant being able to offer you more thorough health checks?	
Yes	15 (68)
No	1 (5)
It depends	6 (27)

^a^Study site was open Wednesday–Friday 11 am–7 pm, Saturday 9 am–5 pm

At the follow-up, of 16 participants asked, six stated that all their attempts to recruit peers were successful, however eight reported unsuccessful attempts to recruit peers (two did not ask anyone), the most common reason given being that peers told them they were “too busy”.

In the focus group, participants noted that the study purpose—to trial new research methods—was well understood, however there was a view this became diluted in subsequent waves and more difficult to communicate. The study location was reported as being accessible by car but less so by public transport. There was a suggestion that the study being located within an HIV testing clinic might have discouraged some people from participating in case they were viewed as being at risk of HIV. Other reasons offered to explain the low response included: low levels of community awareness about health needs disproportionately affecting GBM; lack of community affiliation among peers; general disinterest among peers; the time investment required to travel, complete and recruit peers; and the need to physically transfer the study coupon to peers. Some felt reluctant recommending participation to their peers as the study procedures involved an anal swab.

## Discussion

Our pilot study did not generate the numbers of participants hoped for in the allocated time, indicating that RDS undertaken as we did is unlikely to be an effective or efficient method of generating an adequate sample of GBM living in high income urban gay-friendly settings such as Auckland, New Zealand. RDS studies that offer participants three coupons generally expect a 33 % coupon redemption rate [13]; in our study this was 23 %. However, those who did participate did provide information on their peer network size required for RDS to approximate a random sample, and biological specimens, suggesting aspects of the study are likely to be acceptable in this population. Participants were motivated by altruism above financial incentives, however, time, transport and social factors such as discomfort recruiting friends were perceived as barriers to inviting peers to participate. In addition, two linked participants were identified with undiagnosed rectal chlamydia, highlighting the potential of such a study to estimate STI prevalence among a non-clinic sample of GBM.

The main strength was the multiple feedback pathways used to evaluate this pilot, being the sample size, coupon redemption rate, and three evaluations. Weaknesses are that the study acceptability findings and perceptions of study barriers are by definition limited to responders, which may differ from non-responders.

Several factors may have influenced our pilot study findings. Auckland has a large suburban sprawl and more limited public transport than many cities, making it difficult to physically transfer study coupons from participants to peers, and possibly reducing willingness to visit the study location near the city centre to complete study procedures. Also, New Zealand has a relatively progressive social climate regarding homosexuality [15], potentially diminishing the perceived benefits of study participation and/or raising the threshold required to motivate involvement.

Our respondents indicated that compensation was not the primary motivation for taking part in the study, and indeed research among GBM in New Zealand has a tradition of high participation with minimum compensation [16]. Rather than increasing incentives, reducing the transaction cost for participating in future RDS studies may be more effective in similar settings. Electronic coupons/enrolment have been used elsewhere and could simplify recruitment [17–20]. The behavioural survey could be completed online and biological samples obtained by mailing self-administered kits to participants. However, this potentially creates new risks including verification of a participant’s identity to prevent claiming multiple incentives, difficulty collecting behavioural and biological data synchronously, and study feasibility given budget and technological constraints. More seeds could be recruited once recruitment chains decayed, although this would morph the sampling technique towards a cross-sectional study [20]. Also, participants could be given longer to redeem coupons. Our 2 weeks redemption period is consistent with other RDS studies on GBM however [13] and extending the time would raise staffing costs.

The main implication of our research is that RDS did not prove to be a practicable method for obtaining a useful sample of GBM in Auckland, New Zealand and may not be in similar settings. Refinements and adaptations could improve its feasibility but at a potentially high cost. Instead, efforts to improve sampling and estimates among GBM may be more productively directed towards the inclusion of sexuality questions in general population probability samples, the collection and reporting of sexuality variables in routine health databases, and towards improving non-probability approaches that are already acceptable to GBM. We also highlight the importance of sharing “negative” findings such as these from research piloting new sampling approaches, that will help researchers avoid the costs of implementing RDS in settings where this may prove unfeasible.

## Abbreviations

RDS: respondent-driven sampling
GBM: gay, bisexual and other men who have sex with men
